# Retraction Note to: Loss of PDEF, a prostate-derived Ets factor is associated with aggressive phenotype of prostate cancer: Regulation of MMP 9 by PDEF

**DOI:** 10.1186/s12943-021-01402-x

**Published:** 2021-08-27

**Authors:** Thomas R. Johnson, Sweaty Koul, Binod Kumar, Lakshmipathi Khandrika, Sarah Venezia, Paul D. Maroni, Randall B. Meacham, Hari K. Koul

**Affiliations:** Program in Urosciences, Division of Urology-Department of Surgery, University of Colorado Denver School of Medicine, Denver Veterans Administrative Medical Center, and University of Colorado Comprehensive Cancer Center, Building P15 or RC2, C-317, 12700 E 19th Avenue, Aurora, CO 80045 USA

**Retraction Note to: Mol Cancer 9, 148 (2010)**

**https://doi.org/10.1186/1476-4598-9-148**

The Editor-in-Chief has retracted this article. After publication concerns were raised that the images above the last two bars in Fig. 4D are identified as PC3 cells, whereas in [1] the same images in Fig. 5F above the first and fourth bars are identified as DU145 cells. An investigation by the University of Colorado at Denver was unable to establish the veracity of these images as the original data were not available. The Editor-in-Chief no longer has confidence in the data presented. Hari K Koul, Paul D Maroni, Lakshmipathi Khandrika, Randall B Meacham and Binod Kumar agree with this retraction. Sweaty Koul has not responded to any correspondence from the Editor-in-Chief or Publisher about this retraction. The Editor-in-Chief was not able to obtain a current email address for Thomas R. Johnson and Sarah Venezia.
